# Varying expectancies and attention bias in phobic and non-phobic individuals

**DOI:** 10.3389/fnhum.2013.00418

**Published:** 2013-08-08

**Authors:** Tatjana Aue, Raphaël Guex, Léa A. S. Chauvigné, Hadas Okon-Singer

**Affiliations:** ^1^Swiss Center for Affective Sciences, University of GenevaGeneva, Switzerland; ^2^Department of Psychology, University of GenevaGeneva, Switzerland; ^3^Department of Psychology, University of HaifaHaifa, Israel

**Keywords:** attention bias, biological preparedness, expectancy bias, fear, phobia, spiders

## Abstract

Phobic individuals display an attention bias to phobia-related information and biased expectancies regarding the likelihood of being faced with such stimuli. Notably, although attention and expectancy biases are core features in phobia and anxiety disorders, these biases have mostly been investigated separately and their causal impact has not been examined. We hypothesized that these biases might be causally related. Spider phobic and low spider fearful control participants performed a visual search task in which they specified whether the deviant animal in a search array was a spider or a bird. Shorter reaction times (RTs) for spiders than for birds in this task reflect an attention bias toward spiders. Participants' expectancies regarding the likelihood of these animals being the deviant in the search array were manipulated by presenting verbal cues. Phobics were characterized by a pronounced and persistent attention bias toward spiders; controls displayed slower RTs for birds than for spiders only when spider cues had been presented. More important, we found RTs for spider detections to be virtually unaffected by the expectancy cues in both groups, whereas RTs for bird detections showed a clear influence of the cues. Our results speak to the possibility that evolution has formed attentional systems that are specific to the detection of phylogenetically salient stimuli such as threatening animals; these systems may not be as penetrable to variations in (experimentally induced) expectancies as those systems that are used for the detection of non-threatening stimuli. In sum, our findings highlight the relation between expectancies and attention engagement in general. However, expectancies may play a greater role in attention engagement in safe environments than in threatening environments.

## Introduction

The present study investigates the interplay between two important known biases in phobic and non-phobic fear^1^, namely, expectancy bias and attention deployment bias. Although both have been demonstrated to be core features in phobia, to date, these two phenomena have been investigated independently from each other (expectancy bias: Davey and Dixon, 1996; de Jong and Muris, 2002; Mühlberger et al., 2006; Aue and Hoeppli, 2012; attention bias: Watts et al., 1986; Öhman et al., 2001; Olatunji et al., 2008; Okon-Singer et al., 2011; see also Bar-Haim et al., 2007; Cisler and Koster, 2010; Yiend, 2010, for a review).

Individuals with phobia or extreme fear of specific objects or animals, such as snakes or spiders, exhibit an expectancy bias when estimating the chances of encountering their feared object (de Jong and Muris, 2002; Aue and Hoeppli, 2012). Furthermore, they estimate that once they encounter their feared object, the circumstances will be more negative compared with their own estimations for objects that are less feared by them, and compared with the estimations of non-fearful controls (e.g., Davey and Dixon, 1996; Mühlberger et al., 2006).

Studies on attention bias showed that fearful or phobic individuals tend to engage attention more quickly in their feared stimuli than in unfeared stimuli (e.g., Mogg and Bradley, 2006; Vrijsen et al., 2009); moreover, these individuals are slow in disengaging attention from their feared stimuli compared with unfeared stimuli (e.g., Fox et al., 2001, 2002; Yiend and Mathews, 2001). In addition, fearful or phobic individuals show deficient ability to ignore fear-related distractors compared with non-fearful healthy controls (e.g., Gerdes et al., 2008; Okon-Singer et al., 2011)^2^. Cisler and Koster (2010) suggested that this early vigilance to fear-evoking stimuli is followed by later avoidance (cf. Mogg et al., 1997; Amir et al., 1998; Rinck and Becker, 2006).

Several studies used a visual search task to explore the factors modulating engagement of attention (see review in Yiend, 2010). For neutral items, engagement of attention in certain items presented in a search array has been shown to be modulated by both bottom-up factors, such as color or motion, and top-down factors manipulated via working memory or priming prior to the search (Wolfe et al., 2003; Burra and Kerzel, 2013; Calleja and Rich, 2013; Woodman et al., 2013). A potential origin of biases in attention to threatening material could be biological preparedness (e.g., Öhman et al., 2001). Such preparedness has been hypothesized to increase bottom-up attentional capture (see Yiend, 2010, for details). Little is known, however, about the impact of prior expectancies on attention engagement in fear-relevant targets.

Although quite robust findings have been reported about expectancy and attention biases in fear and phobia, only a single study has so far examined their interrelation, to the best of our knowledge. We (Aue et al., 2013) found evidence that attention and expectancies might be intimately related in spider phobia. Viewing time for spiders in spider phobics was positively related to expectancies for encounters with these animals. Non-fearful individuals, in contrast, displayed a negative association of viewing time and encounter expectancy for spiders. These differential associations between the two groups were, however, unspecific for spiders (i.e., held also for snakes and birds), which can possibly be explained by the generally more stressful nature of the experiment for phobic individuals. Together, these findings suggest that, in potentially threatening situations, there might be a substantial difference in the co-organization of attentional and expectancy processes in phobic and low fearful control participants. Because the study data are of a correlational nature, we were unable to distinguish whether variations in expectancies were at the origin of variations in attention deployment or vice versa.

The current study investigated the directionality of biases in attention and expectancies and tested whether variations in expectancies can cause variations in attention deployment. Despite our earlier focus on visual avoidance (or overall viewing time; Aue et al., 2013), we were now interested in initial attention engagement toward threatening stimulus material. Specifically, we hypothesized that an individual's expectancies concerning frequencies and consequences of confrontations with threatening stimuli could sensitize the individual to certain types of stimuli. Such a top-down mechanism may then lead the individual to engage in active search and may guide his or her attention to evidence in the environment that supports the already existent expectancies (see Krizan and Windschitl, 2007, as well as Aue et al., 2012, for related links in between positive cognitive biases [overoptimism or wishful thinking] and selective attention). Thus, we hypothesized that prior expectancies about the occurrence of threatening events would exert a top-down influence on the visual search for threat (i.e., attention engagement in threat-related targets).

In order to directly examine the effect of expectancies on attentional engagement, we manipulated expectancies regarding the likelihood of different types of targets appearing that were presented in a visual search task. More concretely, spider phobic and low spider fearful control participants in the present study had to search for a deviant spider or bird among eight butterflies. Before the presentation of each search array, a verbal cue informed the participants about the likelihood that the deviant stimulus would be a spider or a bird. By this means, we manipulated our participants' expectancies of encountering (i.e., seeing) spiders and birds.

Three specific hypotheses were tested: First, on the basis of earlier literature (e.g., Bar-Haim et al., 2007), we predicted the attention bias for spiders to be more pronounced in spider phobics as compared with the low spider fearful controls. Second, from the evidence for modulation of detection speed by cueing and predictability in neutral items arrays (e.g., Wolfe et al., 2003; Burra and Kerzel, 2013), we predicted an effect of congruency in that variations in expectancies would modulate detection times in both groups of participants, with expected deviants leading to shorter reaction times (RTs). Third, we hypothesized phobic participants to be less sensitive to variations in externally imposed (or “objective”) expectancies than low spider fearful controls because of the a priori conviction of the former to incur an increased risk to encounter (or detect) spiders (e.g., de Jong and Muris, 2002), even when objective background information regarding the likelihood of an encounter is given (Aue and Hoeppli, 2012).

## Materials and methods

### Participants

Thirty-one participants [16 spider phobic; 8 male (4 in spider phobic group)], aged between 19 and 46 years (*M* = 27.1, *SD* = 6.03) were recruited via ads placed in university buildings and on university and local websites. These ads looked for participants who were either extremely fearful of spiders (including strong physiological response and avoidance), or displayed particularly low fear of these animals. The study was embedded in a larger project investigating decision making, psychophysiological, and central nervous responses while imagining encounters with feared and non-feared animals. The ads explicitly specified these project aims. Persons interested in the study were given a telephone interview and screened with the *Diagnostic and Statistical Manual of Mental Disorders* (4th Edn., Text Rev.; American Psychiatric Association, 2000) and the *International Classification of Diseases* (10th Rev.; World Health Organization, 1992) for criteria for the presence or absence of spider phobia [adapted from Mühlberger et al. (2006)].

Apart from meeting or not meeting criteria for spider phobia, fear of spiders was also assessed by asking the participants to rate their respective fears on a scale from 0 (no fear at all) to 100 (maximal or extreme fear). Spider phobic individuals rated their fear of spiders much higher than low spider fearful control participants did, *t*_(29)_ = 16.06, *p* < 0.000001 (*M*s = 81.4 and 18.7, respectively). Fear of spiders was further assessed after the experiment by the use of the French translation of the Fear of Spiders Questionnaire (Szymanski and O'Donohue, 1995), *t*_(29)_ = 7.32, *p* < 0.000001 (*M*s = 86.2 and 24.3).

### Stimuli

Search array (attention): Stimuli consisted of (a) 30 pictures displaying spiders, all taken from a recently created picture base (Dan-Glauser and Scherer, 2011); (b) 30 birds, collected from the Internet; and (c) 100 butterflies, also collected from the Internet. In each trial, the search array consisted of a matrix of nine different animal pictures with three columns and three rows. There was an equal probability for the spiders and the birds to appear in any of the nine different locations within the matrix. The stimuli were matched for luminance and contrast and displayed in gray scale.

Cues (expectancy): Three different types of cues were presented specifying either “spider 90%,” “bird 90%,” or “spider bird 50%” (for half of the participants; for the other half, the latter said “bird spider 50%”). These cues specified the probability that the to-be detected deviant in a subsequently presented search array would be a spider or a bird.

In reality, the spider 90% (bird 90%) cue condition referred to a probability of 71% (69 trials) that there would actually be a spider (bird) among eight butterflies in the search array presented thereafter. In the remaining cases, either a bird (spider) was presented (23 trials) or no deviant at all (five trials). The latter trials were included to verify that the participants responded on the basis of the target perception.

In the 50% cue condition, there was an equal likelihood that either a spider or a bird would be the deviant in the subsequently shown search array (46 trials; 23 spider deviants and 23 bird deviants). In some cases, there was no deviant (five trials).

### Procedure

Upon the participants' arrival at the laboratory, the nature of the experiment was explained and written informed consent was obtained (protocol approved by the local ethics committee). The experimental task was introduced as a test of the capacity to detect spiders and birds in an array of butterflies. After participants had thoroughly read the task instructions, they performed 10 practice trials to become familiar with the task.

**Figure 1:**
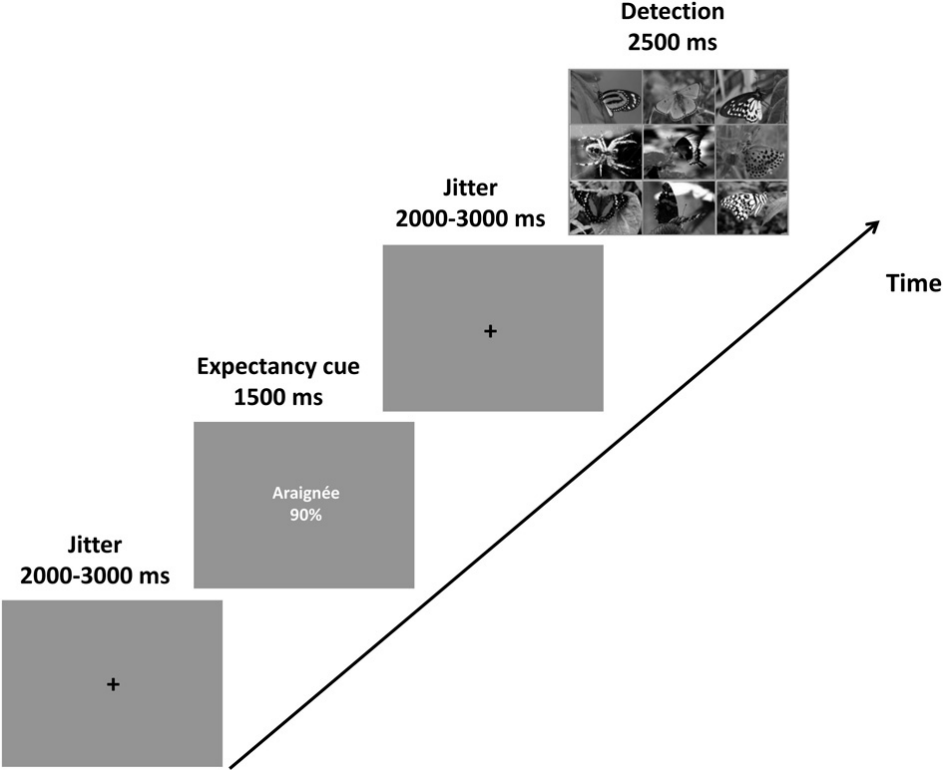
**Task sequence**. An example of a spider-90% cue [araignée (French word for spider) 90%] followed by a search array depicting a spider target. Participants were told that the cues described the likelihood of a spider or a bird being the deviant in the search array. They were asked to respond as quickly and accurately as possible according to the target (i.e., spider, bird, or no target).

Figure 1 shows the sequence of events in an experimental trial. In each trial, participants saw a fixation cross for 2000–3000 ms that was followed by a cue presented for 1500 ms. These cues referred to the probability that the to-be detected deviant in the subsequently presented search array would be a spider or a bird (see preceding section for further details regarding the expectancy cues). After the presentation of the cue, another fixation cross appeared for 2000–3000 ms. Next, the search array, consisting of nine pictures [either nine butterflies (no deviant), eight butterflies and a spider, or eight butterflies and a bird] was shown for 2500 ms. The participants had to decide whether there was no deviant, or whether the deviant was a spider or a bird.

Participants were instructed to react as fast and correctly as possible. Responses were given by pressing three different keys on the computer keyboard; the keys attributed to spiders and birds were counterbalanced across participants. A total of 244 experimental trials were presented in random order [four runs of 61 trials with short pauses in between; the frequencies of trials of different kinds (cues, deviants) were comparable between runs]. The next trial began immediately after the detection period had elapsed. The inter-trial interval was jittered around 9 s.

In a post-experimental questionnaire, the participants specified whether (a) they had paid attention to the cues; (b) it had been easier for them to detect the spider rather than the bird (and the reverse question); and (c) there was a greater risk of a spider rather than a bird being the deviant when the 50% cue had been presented (and the reverse question).

After the participants had completed the Fear of Spiders Questionnaire, they were debriefed.

### Dependent variables

The dependent variables consisted of the participants' RTs for the correct responses. Errors made up ~5% of all responses (*SD* = 3%). We also analyzed differences in expectancies as assessed via the post-experimental questionnaire (see below for details).

### Data analysis

#### Reaction times^3^

A 2 × 3× 2 analysis of variance (ANOVA) with the between-participants factor *group* (spider phobic, low spider fearful control) and the within-participants factors *expectancy* [spider cue (spider 90%), bird cue (bird 90%), ambiguous cue (spider bird 50%/bird spider 50%)], and *target* (spider, bird) was performed on RTs. Significant effects were further investigated by the use of *post-hoc* Tukey tests. An α level of 0.05 (two-tailed) was applied. All reported effect sizes are partial η^2^ and simply noted as η^2^.

The hypotheses led us to expect first a stronger attention bias for spiders (i.e., a greater difference in RTs between spiders and birds) in phobics than in low spider fearful controls, reflected in a significant interaction of the factors group and target. Second, we anticipated an effect of congruency, with detection of spider targets being facilitated by spider cues and detection of bird targets being facilitated by bird cues. Therefore, a significant interaction of expectancy cue and target was predicted. The third hypothesis stated that phobic participants would be less susceptible than control participants to externally induced expectations because of the strong and comparably persistent tendency to expect encounters with spiders in the former group (e.g., de Jong and Muris, 2002; Aue and Hoeppli, 2012). Consequently, we predicted stronger congruency effects in controls than in phobics, as revealed by a significant three-way interaction of group, expectancy cue, and target.

#### Post-experimental questionnaire

Group differences for the questions in the post-experimental questionnaire, to which the participants replied with either “yes” or “no,” were investigated with a χ^2^ test (*df* = 1). An α level of 0.05 (two-tailed) was applied.

## Results

### Reaction times

**Figure 2:**
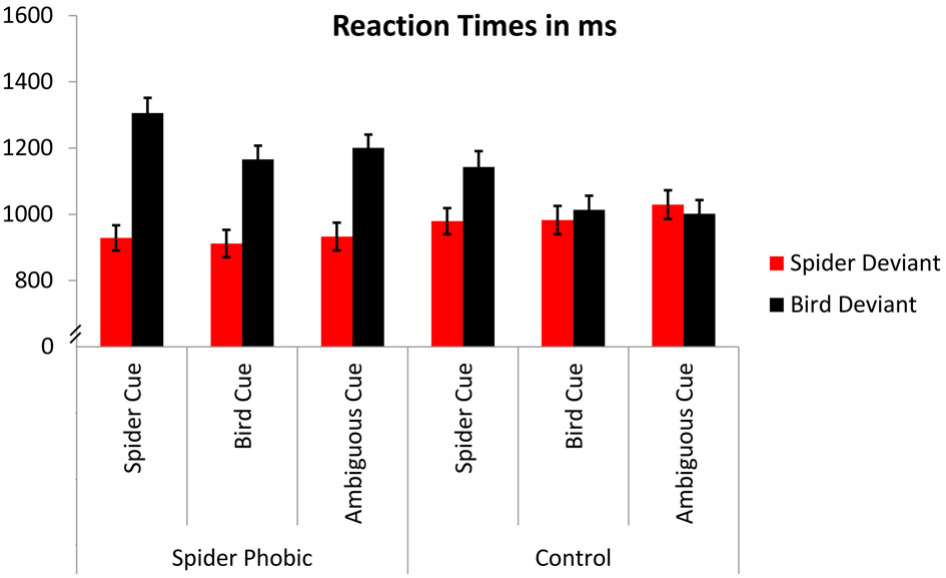
**Reaction times**. Error bars depict standard errors.

The 2 (group: spider phobic, low spider fearful control) × 3 (expectancy: spider, bird, ambiguous) × 2 (target: spider, bird) ANOVA yielded both a significant main effect of expectancy, *F*_(2, 58)_ = 17.76, *p* < 0.000005, η^2^ = 0.38 (*M*s = 1089.2, 1018.4, and 1041.1 ms, for spider, bird, and ambiguous, respectively), and a significant main effect of target, *F*_(1, 29)_ = 37.23, *p* < 0.000005, η^2^ = 0.56 (*M*s = 960.8 and 1138.4 ms, for spider and bird, respectively; see also Figure 2). These effects were qualified by the higher-order interactions described in the following paragraphs.

In accordance with our first hypothesis, in which we predicted a stronger attention bias for spiders being present in phobics compared with controls, the interaction group × target achieved significance, *F*_(1, 29)_ = 17.50, *p* < 0.0005, η^2^ = 0.38 (phobics: *M*s = 924.6 and 1224.0 ms, for spider and bird, respectively; controls: *M*s = 997.0 and 1052.8 ms). Overall, controls did not display different RTs for the detection of spiders and birds (Tukey test for this pairwise comparison: *p* > 0.54), whereas phobics demonstrated particularly slow RTs for the detection of birds (compared with RTs for spiders, as well as compared with RTs for both spiders and birds in the control group; all *p*s < 0.05).

Consistent with our second hypothesis, in which we predicted that expectancy cues would facilitate RTs with respect to congruent targets, the ANOVA revealed a significant interaction of expectancy × target, *F*_(2, 58)_ = 23.24, *p* < 0.000001, η^2^ = 0.44 (spider targets: *M*s = 954.1, 947.2, and 981.0 ms, for spider, bird, and ambiguous cue, respectively; bird targets: *M*s = 1089.7, 1224.4, and 1101.3 ms, for bird, spider, and ambiguous cue, respectively). Somewhat surprisingly, though, expectancy effects were limited to the detection of birds; *post-hoc* Tukey tests for this interaction showed no difference between spider detections related to the three expectancy cues (*p*s > 0.63; all other *p*s corresponding to pairwise comparisons for this interaction < 0.0005).

The interaction of group, expectancy, and target, *F*_(2, 58)_ = 1.79, *p* = 0.18, η^2^ = 0.06, did not reach significance. Nevertheless, on the basis of our third a priori hypothesis that experimentally induced expectancies would modulate detection times in low spider fearful controls more strongly than in phobics, we performed analyses separately for phobics and controls. Phobics displayed a significant main effect of expectancy, *F*_(2, 30)_ = 9.00, *p* < 0.001, η^2^ = 0.37 (*M*s = 1117.2, 1038.8, and 1067.0 ms, for spider, bird, and ambiguous, respectively), due to prolonged RTs for spider cues; a significant main effect of target, *F*_(1, 15)_ = 36.28, *p* < 0.00005, η^2^ = 0.71 (*Ms* = 924.6 and 1224.0 ms, for spider and bird, respectively), due to faster RTs for spider compared with bird targets; and a significant interaction of both factors, *F*_(2, 30)_ = 9.11, *p* < 0.001, η^2^ = 0.38 (spider targets: *M*s = 928.8, 912.0, and 933.0 ms, for spider, bird, and ambiguous cue, respectively; bird targets: *Ms* = 1165.6, 1305.7, and 1200.8 ms, for bird, spider, and ambiguous cue, respectively). Tukey tests revealed that expectancy cues did not differentially influence the detection of spiders (*p*s > 0.93, for the three corresponding pairwise comparisons)^4^. By contrast, expectancy cues clearly influenced the detection of birds in the phobic group, with spider cues leading to slower detections than both bird cues and ambiguous cues (*p*s < 0.001), and no difference between the latter two (*p* > 0.61).

Similarly, in the low spider fearful controls, all effects achieved (or approached) significance: a main effect of expectancy, *F*_(2, 28)_ = 9.34, *p* < 0.0005, η^2^ = 0.40, again due to slowed RTs for spider cues (*M*s = 1061.2, 998.1, and 1015.4 ms, for spider, bird, and ambiguous, respectively); a main effect of target, *F*_(1, 14)_ = 5.13, *p* < 0.07, η^2^ = 0.22, due to faster RTs for spider compared with bird targets (*M*s = 997.0 and 1052.8 ms, for spider and bird, respectively); and an interaction of expectancy × target, *F*_(2, 28)_ = 15.04, *p* < 0.00005, η^2^ = 0.52 (spider targets: *Ms* = 979.4, 982.5, and 1029.0 ms, for spider, bird, and ambiguous cue, respectively; bird targets: *M*s = 1013.7, 1143.1, and 1001.7 ms, for bird, spider, and ambiguous cue, respectively). Tukey tests revealed that the detection of birds was slowed when spider cues had been previously presented (*p*s < 0.005 with respect to all other conditions); no difference was observed between the remaining conditions (*p*s > 0.38). These results demonstrate that, similar to the results in the phobic group, experimentally induced expectancies did not impact RTs for spider deviants in the control group, while they did influence detection of deviant birds.

#### Post-experimental questionnaire^5^

Most phobic and control participants said they had paid attention to the cues (phobics: 12 of 14, controls: 10 of 15; no group difference: χ^2^ = 1.43, *ns*), demonstrating that most of our participants followed task instructions quite well. In line with the RT data, showing a stronger attention bias in phobics compared with controls, most phobics indicated that it had been easier for them to detect spiders rather than birds (9 of 14), whereas less than a third of the control participants thought it had been so (4 of 15), χ^2^ = 4.14, *p* < 0.05. Finally, more phobics than controls specified a greater risk of a spider rather than a bird having been the deviant when the 50% cue had been presented (phobics: 9 of 14, controls: 1 of 15), χ^2^ = 9.96, *p* < 0.005.

## Discussion

First, we had hypothesized that phobic individuals would display a stronger attention bias than controls [cf. meta-analytic data reported by Bar-Haim et al. (2007), and a review of the topic in Okon-Singer et al. (2013)]. Data in the current project are supportive of this hypothesis. We observed a strong and persistent attention bias for spiders in spider phobics. An attention bias for spiders existed also in the low spider fearful control group; however, it was much smaller than in phobics and limited to specific situational requirements: Prolonged RTs for the detection of birds rather than spiders in control participants were observed only when spider expectations had been induced. Such greater context dependency of the attentional bias in controls might explain the inconsistency of results regarding the existence of an attention bias for threatening animals in healthy individuals (positive findings: e.g., Öhman et al., 2001; Lipp and Waters, 2007; null findings: e.g., Tipples et al., 2002; Lipp et al., 2004).

It is noteworthy that, in contrast to Bar-Haim et al.'s (2007) meta-analytic data, the difference between spider phobic and low spider fearful control participants in the current study was not so much based on the particularly rapid RTs of spider phobics for spiders, but more strongly on the particularly slow RTs of these participants for birds. It is possible that the phobic participants did not trust the bird cues, and due to an a priori expectancy bias (e.g., de Jong and Muris, 2002; Aue and Hoeppli, 2012) that was independent from our experimental manipulations, expected the spiders with a higher likelihood than birds. Spider phobics may have therefore started to selectively and quickly scan the visual array for spiders in all trials. Such biased stimulus processing may have impeded attention engagement in birds until it had been determined that there really were no spiders. Support for such an idea comes from the responses of the phobics to the post-experimental questionnaire, in which they even retrospectively specified a greater risk of a spider rather than a bird having been the deviant when the 50% cue had been presented. The fact that participants were told that spider and bird cues indicated a likelihood of 90% for the cued animal to be the deviant target in the visual search array, but the actual likelihood was only 71%, may have further increased the distrust in the cues.

Second, we had predicted an effect of congruency in that variations in the experimentally induced expectancies would modulate attention engagement in both groups of participants, with expected deviants leading to a shortening of RTs and unexpected deviants leading to a slowing of RTs. Such an effect was demonstrated earlier for search arrays presenting neutral items (e.g., Wolfe et al., 2003; Burra and Kerzel, 2013). Burra and Kerzel (2013), for instance, examined attentional capture during a visual search task while varying the predictability of neutral targets. Their findings show that predictability influenced attention engagement by modulating the search mode (singleton vs. feature search). In line with these results, we had predicted that prior expectancies given by the cues would modulate attention engagement in the current study.

In a general sense, we were indeed able to show that experimentally induced “objective” expectancies can impact RTs related to the detection of deviant animals in a visual search array. These findings hence support the existence of a causal link between expectancies and attention engagement. However, contrary to our predictions, this link is not simple: Our ANOVA results suggest that the experimentally manipulated expectancies did not influence attention deployment for the detection of spiders in both the spider phobics and the low spider fearful controls. Yet, replicating earlier results for neutral stimulus material (e.g., Burra and Kerzel, 2013), the experimentally induced expectancies—specifically the spider cues—had an impact on the detection of birds. Thus, the deviant needed to be neutral for an increase in RTs to be detected in the invalid trials. This pattern of results suggests that the detection of threatening stimuli relies on different mechanisms than those required for the detection of neutral stimuli.

The modulation of attention capture by cues shown for bird targets is in line with models of visual detection that emphasize the role of priming and working memory representations in the modulation of visual search. For example, according to the attentional engagement theory (Duncan and Humphreys, 1989, 1992), attentional selection is modulated by templates actively maintained in memory. Similarly, the guided search model (Wolfe, 2003, 2010; Wolfe et al., 2003) argues that an a priori map guides subsequent search behavior. This top-down attention guidance may be manipulated via explicit task demands, or implicitly via expected target identity created through priming. Together, these theories suggest that if a sensory input matches a set of predefined properties, it will lead to involuntary shifts of attention. The results for birds in our study suggest that our experimental manipulation of expectancies may have influenced the content of such an a priori map (i.e., set of predefined properties), but these manipulations clearly did not affect attention engagement to threatening information.

That there was no congruency effect for spider targets in phobics can be possibly explained by the fact that phobic participants are characterized by generally increased a priori expectancies of being presented with images of spiders (e.g., de Jong and Muris, 2002; Aue and Hoeppli, 2012). The experimentally induced expectancies in the present study may have influenced these habitual encounter expectancies only slightly, or even not at all, thereby being ineffective in producing significant changes in the habitually increased vigilance for spiders in spider phobics. If this effect were observed in phobics but not in controls, the data would be consistent with our third hypothesis that stated that phobic participants would be less sensitive than controls to variations in externally imposed (or “objective”) expectancies.

Yet, contrary to our predictions, experimentally induced expectancies did not influence RTs for spiders in controls either, and these participants are not generally characterized by increased expectancies of encounters with spiders (Aue and Hoeppli, 2012). Therefore, our data do not allow the conclusion that an external induction of expectancies regarding spider encounters is generally more successful in non-fearful control participants than in phobic participants. That both groups of participants were able to follow task instructions and adopt different expectancy states according to the cues presented, however, is proven by the participants' differentiated responses to birds. Hence, the observed null finding for an influence of expectancies on spider detection speaks to specialized attention engagement toward threatening information.

Diverse kinds of specialized attention engagement in threatening information have been reported before (Öhman et al., 2001; Notebaert et al., 2011; see review in Yiend, 2010). Öhman et al. (2001) showed faster detection of threatening animals compared with flowers and mushrooms in non-phobic individuals. Interestingly, this quicker detection was amplified in phobic individuals [contrary to the current study, but in line with Bar-Haim et al.'s (2007), review; for a discussion of these inconsistencies, see first hypothesis above]^6^. Because an attention bias was found for phobic and non-phobic participants, the findings were interpreted as a preattentive prioritization of threat information due to biological preparedness. However, albeit the observation that detection of fear-relevant animals is prioritized, it has been shown that the degree of facilitation depends on the number of distracting items in the search array, thus contradicting the idea of preattentive processing of threatening stimuli (Batty et al., 2005; Notebaert et al., 2010, 2011).

Our findings are consistent with the view of a universal (i.e., not linked to high levels of fear) evolutionary heritage for the processing of threat^7^. This view may explain why the detection of evolutionary salient stimuli such as threatening animals (here: spiders) is not as penetrable to experimental expectancy manipulations as the detection of non-threatening stimuli (for similar findings using a dot-probe task in unselected participants, see Lipp and Derakshan, 2005). Such an evolutionary mechanism would be in line with the idea of biological preparedness for certain classes of stimuli (Seligman, 1971; Öhman and Mineka, 2001) and would ensure quick adaptive behavioral responses in the service of survival (e.g., Flykt et al., 2012), responses that can be initiated without requiring adequate expectancy states.

It has been proposed that fear responses can be rapidly mediated by a network of subcortical structures (the so-called fear module, including, for instance, the amygdala; Lang et al., 1998; LeDoux and Phelps, 2000; Davis and Lang, 2003). The amygdala is capable of initiating a defense response via connections to the hypothalamus and the brainstem, even without conscious processing of information regarding a threatening stimulus. Therefore, and because of the persistence of phobias despite the explicit knowledge that a feared object is not harmful, the fear module has been proposed to be “impenetrable to conscious cognitive control” (Öhman and Mineka, 2001, p. 515). The fear module may hence ensure automatic processing of survival-relevant stimuli that is independent of explicit (or externally imposed) expectancies.

However, it is also possible that the spider pictures in our study popped out due to a common physical characteristic (e.g., curved line body with eight legs). This could alternatively explain why spiders were in general detected very fast and why cue manipulation did not affect RT for spider pictures in both groups. Birds may lack such a pop-out characteristic. As a consequence, our cues may have affected the detection of birds only. Note, however, that fast capture of attention by stimuli associated with threatening animals was previously shown even when visual features had been controlled for. Batty et al. (2005) used a visual search task with conditioned stimuli. Participants with high or low fear of spiders or snakes detected abstract shapes that had been paired earlier with either a neutral picture or a picture of their feared animal. In this study, both high and low fearful participants were overall faster at detecting targets that were associated with negative animals.

In line with the authors' conclusion that visual features are not the reason for facilitated capture of attention by threatening items, we do not think that differences in visual features can fully explain the effects found in the current study. Two additional reasons further strengthen our conviction on this issue. First, the butterfly distracters in our study were selected on the basis of the assumption that they share significant features with both spiders *and* birds (e.g., wings corresponding to birds' wings; six legs + two antennas corresponding to the eight legs of spiders). About 75% of the butterflies were displayed from their side, clearly showing all legs and antennas. All images were displayed in gray scale, preventing pop-out effects based on color differences between stimulus categories. Second, if pop-out effects had been responsible for our effects, controls should have displayed an overall greater discrepancy of RTs for spiders and birds.

Subsequent studies should test for the existence of an influence of attentional processes on expectancies, for instance by manipulating vigilance to or avoidance of threat. Deviations in attention (e.g., selective visual attention) may lead to biased expectancies about future outcomes because reality is experienced in specific or selective ways. Adding eye-tracking and event-related potentials to the research tool inventory might help to identify basic mechanisms underlying phobia-related processing biases, their time course, and their interdependence. Neuroimaging may further increase knowledge by directly examining the impact of the suggested subcortical regions (e.g., in the so-called fear module; Lang et al., 1998; LeDoux and Phelps, 2000; Öhman and Mineka, 2001; Davis and Lang, 2003).

It is also important to note that our control group displayed particularly low fear of spiders and therefore might be characterized by specific responding. We cannot rule out that some of the control participants specifically liked these animals, because we did not assess independent data on the pleasantness or appeal of the animals in the current study. The question of whether participants characterized by “normal” fear of spiders exhibit the same pattern of response as phobics and low fearful controls remains to be investigated. Another aspect that should be examined is whether the effects observed by us can be reproduced for threatening stimuli other than animals that have been proven dangerous throughout evolution (e.g., guns and knives) in order to test the biological preparedness account. Finally, future studies are needed to rule out the possibility that the differences in RTs we observed in the current study are related to motor processes rather than attention engagement.

In conclusion, both phobics and low spider fearful controls showed an attention bias and less influence of expectancy cues in the detection of spiders, in line with the biological preparedness view. However, the attention bias was larger in phobics, whereas it was restricted to specific expectancy conditions (i.e., spider cues, which produced a slowing of the detection of bird targets) in controls. Taken together, our results highlight the relation between expectancies and attention engagement in general. However, the influence of expectancies may be inhibited during the processing of threatening stimulus material, and expectancies may play a greater role in safe compared with threatening environments. Thus, at first glance, our data challenge the hypothesis that expectancy bias is at the origin of attention bias. Nonetheless, we cannot rule out that our participants exhibited a priori expectancy biases (leading to a preferential processing of spider-related targets) and that these biases were too strong to be overruled by the expectancy cues used in the present study. Future research should eliminate this possibility before safe conclusions can be drawn. In general, investigating the mutual relations between expectancy and attention biases may lead to a more comprehensive model of processing of threat in health and anxiety disorders. Notably, although attention and expectancy biases are core features in phobia and anxiety disorders, these biases were mostly investigated separately and their causal impact has not been examined. The current findings add much needed data to this emerging field of combining different types of bias. Such an approach may lead to therapeutic approaches that are more effective than selective targeting of either attention or expectancy bias.

### Conflict of interest statement

The authors declare that the research was conducted in the absence of any commercial or financial relationships that could be construed as a potential conflict of interest.

## Footnotes

^1^We refer to individuals with extreme fear, but who were not clinically diagnosed as phobic, as “fearful.” Phobic individuals in the context of the present paper refer to individuals who were clinically diagnosed.

^2^A meta-analysis (Bar-Haim et al., 2007) revealed that attention bias is consistently found when stimuli are presented outside awareness. This suggests that the bias for threat-related material in fearful and anxious participants originates at an early subconscious stage. Yet, part of it may also rely on conscious processing and top-down influences because larger effect sizes are generally observed when participants are aware of the presented stimulus material (Bar-Haim et al., 2007).

^3^We also performed analyses on logarithmic RTs and excluded outliers (±3 *SD* from individual average RT). However, effects observed in the current study were not affected by these transformations of the data. Therefore, results for only the original data will be described.

^4^But detection of spiders was altogether more rapid than detection of birds (all *p*s < 0.0005), reflecting a strong and persistent attention bias to fear evoking compared with neutral information in phobic participants.

^5^As a result of time limitations, two phobic participants did not complete the post-experimental questionnaire.

^6^Note as well that there were significant differences between the experimental paradigms used in these earlier studies and our own. For instance, the earlier studies did not use expectancy cues.

^7^As outlined above, such processing is not necessarily preattentive.
